# The Treatment of Piles

**Published:** 1884-01-20

**Authors:** William H. Veatch

**Affiliations:** Carthage, Ill.


### THE TREATMENT OF PILES.

By WILLIAM H. VEATCH. M. D., CARTHAGE, ILL.

Since the publication of my articles on this subject in the July and August numbers of the Monthly, I have been completely overwhelmed with letters from members of the profession—representing ten States—asking for more light on the subject of the treatment of piles. These missiles were so numerous that I found to answer them personally would be too great a tax on my time, so I concluded to answer them all through the columns of The Monthly.

I have received no communication taking issue with my theory or plan of treatment, but in the October number of the Monthly Dr. Allen, of Jerseyville, who opened such a vigorous bombardment of our works in May last, and who claimed to have routed the whole camp of the hypodermic army, has been heard from again. This time he shows no disposition to renew the battle, according to the established rules of honorable warfare, but has undertaken the bushwhacking plan.

His article is really amusing, and at the same time supremely ridiculous. What he is pleased to call his "criticism" causes much such feelings as were aroused in the bosom of a young lass whose passionate lover stood before her with hands clasped and eyes glistening with emotion, his whole frame trembling, as he said: "Susanner, only think ! I have soared around like a lone trochilus, flitting from flower to flower, from sweet to sweet, and at last have presented myself before a blushing rose, from which I hope to draw my life long sustenance. If I could but convince you that you are my only teraphine, I would assume the pinions of the oriental zephyrs of the boreal pole, and pluck the last feather from the sheltering wing of the noble bird of personal liberty, as he sits perched on the topmost branch of the magnificent gum tree from which I draw my daily subsistence."

"Susanner, to gain one smile from your ruby lips, and realize that the firmamental azure of your lustrous orbs is fixed on so insigni ficant a thing as me, I would pass through flood and flame, and even penetrate pandemonium. Yes, I would shed——would shed ——w-o-u-l-d—s-h-e-d——."

"Never mind the wood-shed," said she, "go on with your pretty talk."

The doctor, in his extreme anxiety to make a "point," pretends to quote me, and in the twenty-eighth line he quotes he makes only twenty-two mutilations in wording and punctuation, putting periods in the middle of sentences and supplying the word "haven't" for "having" and "would" for "could," and many other mutilations unworthy fair and honest disputation.

One is reminded of the lines of Swift:

> "A strong dilemma in a desperate case,
> To act with infamy, or quit the place."

The principal interrogatories of these correspondents asking for information on this subject may be found below, with my answers attached to each. If I have overlooked none, they appear in the following order:

Question. What is your mode of examination in piles?

Answer. I confine myself to two principal modes of examination: 1st. The knee-breast position of the patient, placed on a table two by six feet, well cushioned. My stand is taken on the left side of the patient. Pressing the nates apart will reveal any external tumors which exist; or the finger may be introduced through the sphincter ani to explore for internal tumors. 2d. I place the patient on the table, on the left side, the limbs flexed on the body, the right limb being drawn higher than the left. with the knee resting on the table, then make the examination as before.

Q. Do you regard internal and external piles of the same nature, and requiring the same treatment?

A. Yes. Piles originate from a common cause; *i. e.*, obstruction of the hemorrhoidal veins, therefore they are of the same nature and may be cured by the same treatment.

Q. What make of syringe do you use?

A. The ordinary hypodermic syringe of Tiemann & Co., is the one I have always used.

Q. How do you manage the needle? At what point do you deposit the remedy, at the apex, center or base of the tumor?

A. The management of the needle is an easy matter when your patient is in proper position and the tumors properly exposed. Use due caution in filling the syringe; see that no air is left in the barrel; insinuate the needle gently into the tumor at any point from which you can most easily reach the sack, or center of the tumor. I have sometimes thought I have ha l better results from depositing the remedy at the base of the tumors, but in so doing I am aware that I risk depositing the fluid in the cellular tissue beyond the hemorrhoidal tumor, or in an unobstructed vessel beyond the limits of the tumor. In such an event I can easily see how we might realize Dr. Allen's fears of embolism. The safest plan, therefore, is to pierce the tumor at its apex or center.

Q. What is the strength of your solution ?

A. I have used all strengths, from equal parts of carbolic acid and water (See report of Typical Case No. 4, in August number of Monthly) to that of only five per cent. of acid, and have had good results from all ; but as a rule, I use a twenty-five per cent. solution. Patients will bear this strength, as a rule, without complaining.

Q. Do you use any other than carbolic acid solution in the treatment of piles ?

A. Yes ; I have used tr. iodine, sol. sub. sulph. ferri, tr. ferri chlor., sol. plumbi acetat, sol. zinci sulph. and simple cold water. Anything that will coagulate the blood. Several of the above act more promptly in that way than carbolic acid, but my experience is in favor of the acid on account of the readiness with which absorption takes place after its use. A little alcohol thrown into the tumor after the coagulation has formed will assist absorption.

Q. What is the exact formula which you have found most suitable, and least painful in the treatment of both internal and external piles ?

Ans. :

| | |
|---|---|
| Acid carbolic........................ } | aa. 1 fl. ounce, |
| Glycerini............................ } | |
| Morphinæ sulph........................ | 8 grains, |
| Aquæ dist............................. | 2 fl. ounces. |

M. Sig. Inject from five to ten drops into each tumor once in two weeks.

Q. Do you inject more than one tumor at each treatment ?

A. In nervous persons, who are easily hurt, and complain of very slight causes of pain, I inject but one at a time. But frequently I inject all at once. if there are half a dozen, (as in the case of No. 2 in report of typical cases.)

Q. What is the difference in pain in internal and external piles ?

A. External tumors are always much more painful under the operation, and are much longer being absorbed.

Q. Do you ever have ulceration follow the injection of carbolic acid ?

A. Occasionally tumors suppurate and discharge considerable quantities of pus. just as they frequently do without an operation of any kind ; but these pus sacks usually granulate and heal with but little difficulty.

Q. How long does it take to cure a case of piles, either internal or external ?

A. I give great latitude in this regard. They have run all the way from five days to five months. A great deal depends on the length of time the tumors have existed.

Q. Do the tumors ever return after being cured by your plan ?

A. No. When the tumor is once cured the vein at that point is obliterated and cannot fill again ; but obliterating the vein at one point will not prevent a tumor from forming in any other part of the vein.

Q. What length of time do patients suffer after treatment ?

A. This depends in a great degree on the condition of the tumor, the sensitiveness of the patient, and the strength of the solution used. Ordinarily the first twelve hours puts an end to the pain, *i. e.*, the pain consequent upon the treatment.

Q. What instruments are necessary for the examination and treatment of piles ?

A. The finger and eye are all you want for an examination of any case. A two-valve speculum, a tenaculum and scissors, a camel's hair pencil and a sponge are all the instruments you will require, besides your syringe, to treat any case of true hemorrhoids.

Q. Can you cure piles and allow your patients to go about their ordinary work ?

A. I can now call to mind only two cases who went to bed in consequence of the treatment. Almost all say that the pain of treatment is not to be compared to the pain they have suffered during the inflammatory stage of the recently filled tumors.

These are the principal questions I have been able to cull from the mass of letters I have received, and I have to regret that my space will not allow me to enter more fully into the discussion of the various topics presented by my correspondents.

Now, I will say to one and all, that the disease is to be treated as all other diseases must be, by the expenditure of a good proportion of common sense, and if one does not understand it he had better keep hands off. Always remember Prof. Andrews' admonition. "This nor no other plan is exempt from danger, when practiced by ignorant men."

First, understand the nature of the parts diseased. Second, understand the disease you are attempting to cure. Third, understand the nature of the remedy you are making use of ; and, fourth, understand how to apply them. With these simple rules in view one can scarcely do harm.—*Peoria Med. Monthly.*

